# Research progress in gene editing of Brassicaceae crops

**DOI:** 10.1016/j.bidere.2026.100122

**Published:** 2026-09-28

**Authors:** Xiaolong Wang, Bingbing Shangguan, Yanwen Zhang, Tongbing Su, Jialei Ji, Jianjun Zhao, Changwei Zhang, Ying Li, Xilin Hou, Tongkun Liu

**Affiliations:** a Key Laboratory of Biology and Genetic Improvement of Horticultural Crops (East China), State Key Laboratory of Crop Genetics & Germplasm Enhancement, Ministry of Agriculture and Rural Affairs of China, Engineering Research Center of Germplasm Enhancement and Utilization of Horticultural Crops, Ministry of Education of China, Nanjing Agricultural University, Nanjing, 210095, China; b Beijing Vegetable Research Center (BVRC), Beijing Academy of Agriculture and Forestry Sciences, Beijing, 100081, China; c State Key Laboratory of Vegetable Biobreeding, Institute of Vegetables and Flowers, Chinese Academy of Agricultural Sciences, Beijing, 100081, China; d College of Horticulture, Hebei Agricultural University, Baoding, 071001, China

**Keywords:** Brassicaceae crops, Genome editing, CRISPR-Cas systems, Genetic improvement, Agronomic traits

## Abstract

Brassicaceae crops are globally important economic plants, whose yield and quality directly affect the supply of edible oil and vegetables. Most of these crops are polyploids with complex genomes and numerous homoeologous gene copies, which demand high efficiency and specificity from gene editing tools, while providing ideal models for studying the rules of polyploid genome editing. Traditional breeding methods, such as crossbreeding and mutation breeding, are constrained by long cycles, low efficiency, and poor specificity, making them insufficient for precise genetic improvement. Gene editing technologies, characterized by precision, efficiency, and targeting specific genomic loci, are core tools for crop breeding. Among these, ZFNs and TALENs rely on protein-DNA recognition and are complicated and costly to design; CRISPR-Cas, which achieves precise recognition through base pairing between guide RNA and target DNA, is simple, efficient, and the most widely used editing tool. Base and prime editors enable single-nucleotide substitutions or small fragment insertions without generating double-strand breaks, making them suitable for directed modification of point mutations. This review summarizes the progress of gene editing in Brassicaceae for bolting/flowering, plant architecture, nutritional quality, tolerance to abiotic stresses (temperature, salinity, drought, heavy metals), and resistance to biotic stresses (diseases, pests). We analyze challenges, including off-target effects, difficulties in polyploid genome editing, and imperfect genetic transformation systems, and discuss future directions integrating gene editing with multi-omics and artificial intelligence. This review aims to provide a theoretical basis and technical support for precise molecular breeding of Brassicaceae crops.

## 1 Introduction

Brassicaceae vegetables are among the most widely cultivated vegetable crops globally, encompassing approximately 357 genera and 4158 species. They are predominantly distributed in the northern temperate zone, with cultivated or wild populations found on all continents except Antarctica [1]. The Brassicaceae family is vast; statistics indicate that the annual global cultivation area of Brassicaceaae crops exceeds 40 million hectares, with their output value accounting for over 35% of the total vegetable output value. They play an irreplaceable role in ensuring food security, promoting rural economic development, and sustaining agroecosystem balance [2]. Brassicaceae crops are rich in various nutrients such as β-carotene, anthocyanins, vitamin C, vitamin B2, folic acid, and dietary fiber, which are vital for enhancing nutritional value to maximize both production and economic benefits. Relevant studies have demonstrated that glucosinolates are a major class of sulfur-rich secondary metabolites that are nearly ubiquitous and largely restricted to Brassicaceae species. Their hydrolytic derivatives, especially isothiocyanates, exhibit prominent anticancer bioactivities and well-documented chemopreventive properties [3,4].

As the fundamental basis for the improvement of plant traits, genetic variation is central to plant breeding, which focuses on generating and deploying elite genetic variation resources [5]. However, traditional breeding is constrained by environmental factors, is time-consuming and labor-intensive, and exhibits low selection efficiency; consequently, modern crop improvement has increasingly shifted towards biotechnology-based breeding. Genetic modification represents a major breakthrough in plant breeding, greatly expanding genetic variation; however, to date, only a small number of genetically modified crops have been utilized. As this technology involves the random integration of exogenous genes into the plant genome, these genetically modified organisms (GMOs) are subject to strict regulation; the commercialization of GM products is constrained by costly and complex regulatory assessment processes, as well as concerns over public acceptance [6]. Different from transgenic breeding that relies on exogenous gene integration and causes uncertain genomic perturbations, gene editing enables precise modification of crop endogenous genomes without foreign gene insertion, and its induced variations are highly analogous to natural mutations with improved biosafety. Since 2022, China has implemented differentiated supervision policies, exempting gene-edited crops without exogenous residues from strict transgenic regulation, which effectively promotes the development of multi-omics and AI-assisted precision breeding as well as germplasm innovation [7]. In recent years, genome editing technologies have developed rapidly; consequently, the precise and efficient modification of plant genomes through genome editing is of significant importance for advancing the genetic improvement of Brassicaceae crops. Plant genome editing is primarily achieved using programmable sequence-specific nucleases (SSNs), which primarily include engineered homing endonucleases (meganucleases), zinc-finger nucleases (ZFNs), transcription activator-like effector nucleases (TALENs) and CRISPR-Cas systems. These nucleases recognize and cleave specifically at target sites in the genome, inducing double-strand breaks (DSBs) in the DNA, which are subsequently repaired via endogenous cellular DNA repair mechanisms, thereby enabling precise and efficient targeted genome modification [8]. Genome editing technologies have been developed to introduce precise and predictable genomic modifications to obtain desired traits, and these technologies are driving the next generation of precision plant breeding [9]. Genome editing technologies hold great potential for the development of new varieties of Brassicaceae crops that are resistant to pests and diseases, tolerant to environmental stresses, high-yielding, and of high nutritional quality.

## 2 Overview of plant gene editing technologies

### 2.1 Conventional gene editing technologies

#### 2.1.1 Zinc-finger nucleases (ZFNs)

To achieve precise genome editing, various artificial nucleases have been developed sequentially. Zinc-finger nucleases (ZFNs) represent the first generation of genome editing tools; comprising two functional modules: a tandem array of zinc-finger (ZF) DNA-binding domains and the non-specific cleavage domain of the FokI restriction endonuclease. Their discovery originated from biochemical studies on the interaction between the African clawed frog (*Xenopus laevis*) transcription factor IIIA and 5S rRNA [10]. As shown in the structural diagram of ZFNs in Fig. 1, the zinc-finger domain is a self-contained structural unit, in which a single zinc ion is chelated by a pair of cysteines and a pair of histidines to chelate a single Zn^2+^ ion in a tetrahedral coordination geometry, resulting in a structurally stable complex. Zinc-fingers can be arranged in a linear series to recognize nucleic acid sequences of varying lengths; by fusing tandem zinc-fingers arrays to nucleases or integrases to form chimeric proteins, the genome modification can be achieved [11]. They are commonly used for targeted gene knockout. The principle involves using ZFNs to induce double-strand breaks (DSBs) at the target site; in the absence of a homologous donor template, the DSB is repaired through the natural process of non-homologous end joining (NHEJ). During this repair process, insertions or deletions (indels) frequently occur at the break site, leading to frameshift mutations and the generation of loss-of-function alleles that abrogate the function of the protein product.

**Fig. 1:**
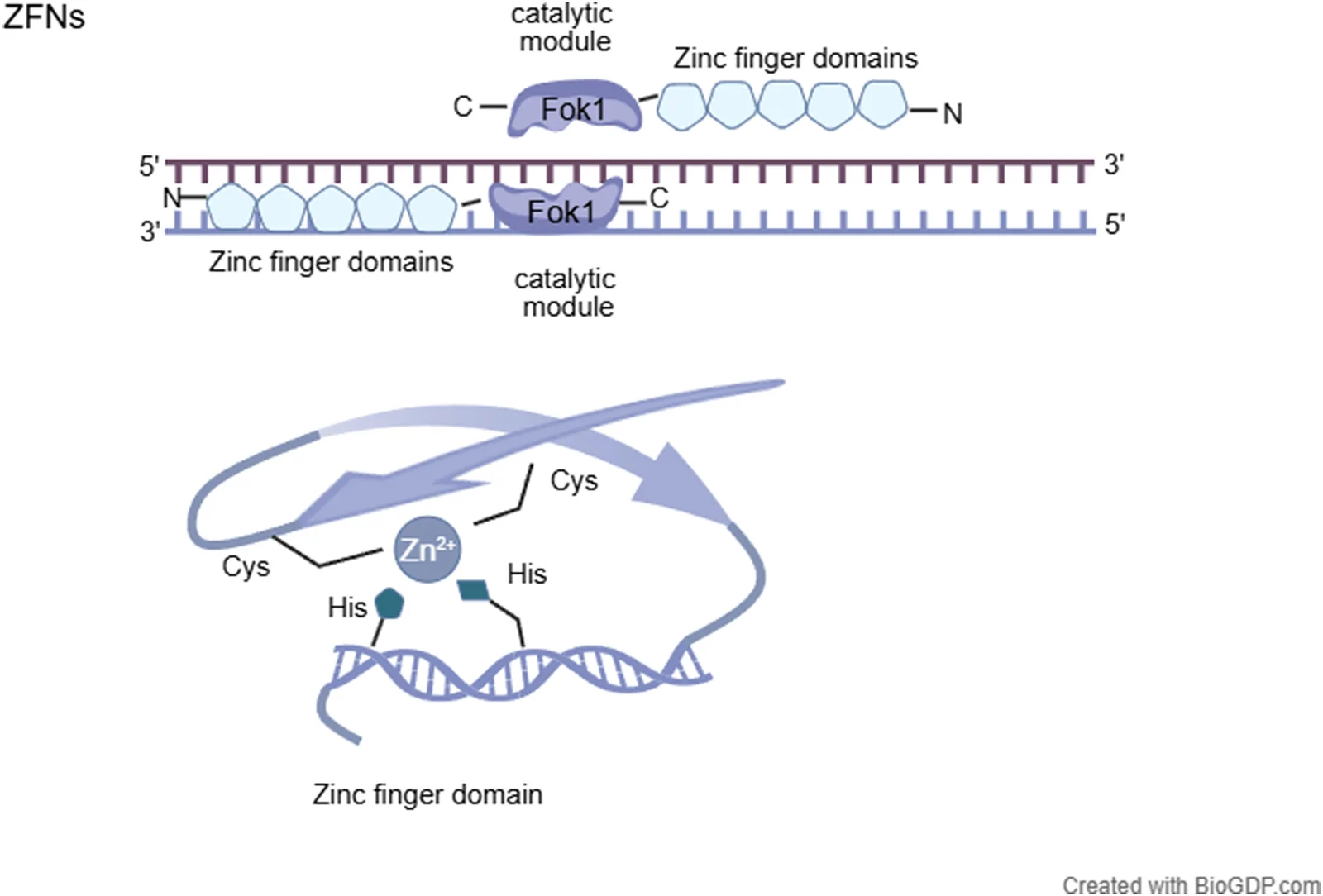
Schematic illustration of the structure and mechanism of Zinc Finger Nucleases (ZFNs). (Top panel) A ZFN is a chimeric endonuclease composed of an N-terminal zinc finger DNA-binding domain and a C-terminal FokI catalytic cleavage domain. Two ZFN monomers bind specifically to adjacent target sequences flanking a short spacer region, which promotes the dimerization of the FokI domains to introduce a site-specific double-strand break (DSB). (Bottom panel) The magnified inset provides a detailed view of a single zinc finger domain interacting with DNA. It demonstrates the classical Cys2-His2 motif, in which two cysteine (Cys) and two histidine (His) residues coordinate with a zinc ion (Zn^2+^) to stabilize the protein's alpha-helix structure, allowing for sequence-specific recognition and binding within the major groove of the DNA helix. Abbreviations: ZFN, zinc finger nuclease; Cys, cysteine; His, histidine; Zn^2+^, zinc ion.

ZFNs are widely used in the field of plant gene editing and were the mainstay of early gene editing. For example, researchers first validated the feasibility of zinc-finger nuclease (ZFN)-mediated targeted mutagenesis in *Arabidopsis thaliana*, laying a crucial foundation for precision editing of plant genomes and clarifying the mutagenic efficiency, mutation types and genetic transmission characteristics of ZFNs in plants [12]. The relevant team adopted ZFNs designed with the OPEN platform alongside an estrogen-inducible expression system and induced frequent mutations in the *ADH1* and *TT4* genes of *Arabidopsis thaliana*. [13]. Other research teams at the same time utilized modularly assembled ZFNs to successfully target and knock out the *Arabidopsis ABI4* gene, yielding stably heritable mutants with ABA and glucose insensitivity phenotypes, together with the concurrent research by Feng Zhang's team, this demonstrated the feasibility of ZFN technology in plants, marking the formal beginning of the era of precision genome editing in plants [14]. ZFN-mediated manipulation enabled targeted deletion, inversion and large chromosomal excision of tandem gene clusters in *Arabidopsis thaliana*, with fragment sizes ranging from 4.5 kb to 9 Mb. This breakthrough resolved a major obstacle that had long hindered functional studies on plant tandem duplicated genes [15]. Although ZFNs represent a groundbreaking technological advance, the design of their recognition domains is complex, requiring the customization of modular zinc-finger protein combinations for each target site. This results in a cumbersome construction process, high technical barriers and poor versatility.

#### 2.1.2 Transcription activator-like effector nucleases (TALENs)

Transcription activator-like effector nucleases (TALENs) are chimeric nucleases composed of DNA-binding domains derived from transcription activator-like effectors (TALEs), which are a class of proteins naturally expressed by certain plant-pathogenic bacteria [16]. The DNA-binding domain consists of multiple tandemly repeated motifs, each recognizing a single nucleotide within the target DNA sequence. The amino acid composition of these repeats varies, with differences in the 12th and 13th amino acid residues being particularly critical, as they directly determine the binding specificity of the TALE–DNA interaction. The modular nature of these repetitive regions enables the precise targeting of virtually any DNA sequence [17]. As shown in Fig. 2 these effectors can be engineered to recognize and bind specific DNA sequences, while the FokI nuclease domain introduces double-strand breaks at the target site, facilitating precise genome editing via non-homologous end joining (NHEJ) or homology-directed repair (HDR) [18].

**Fig. 2:**
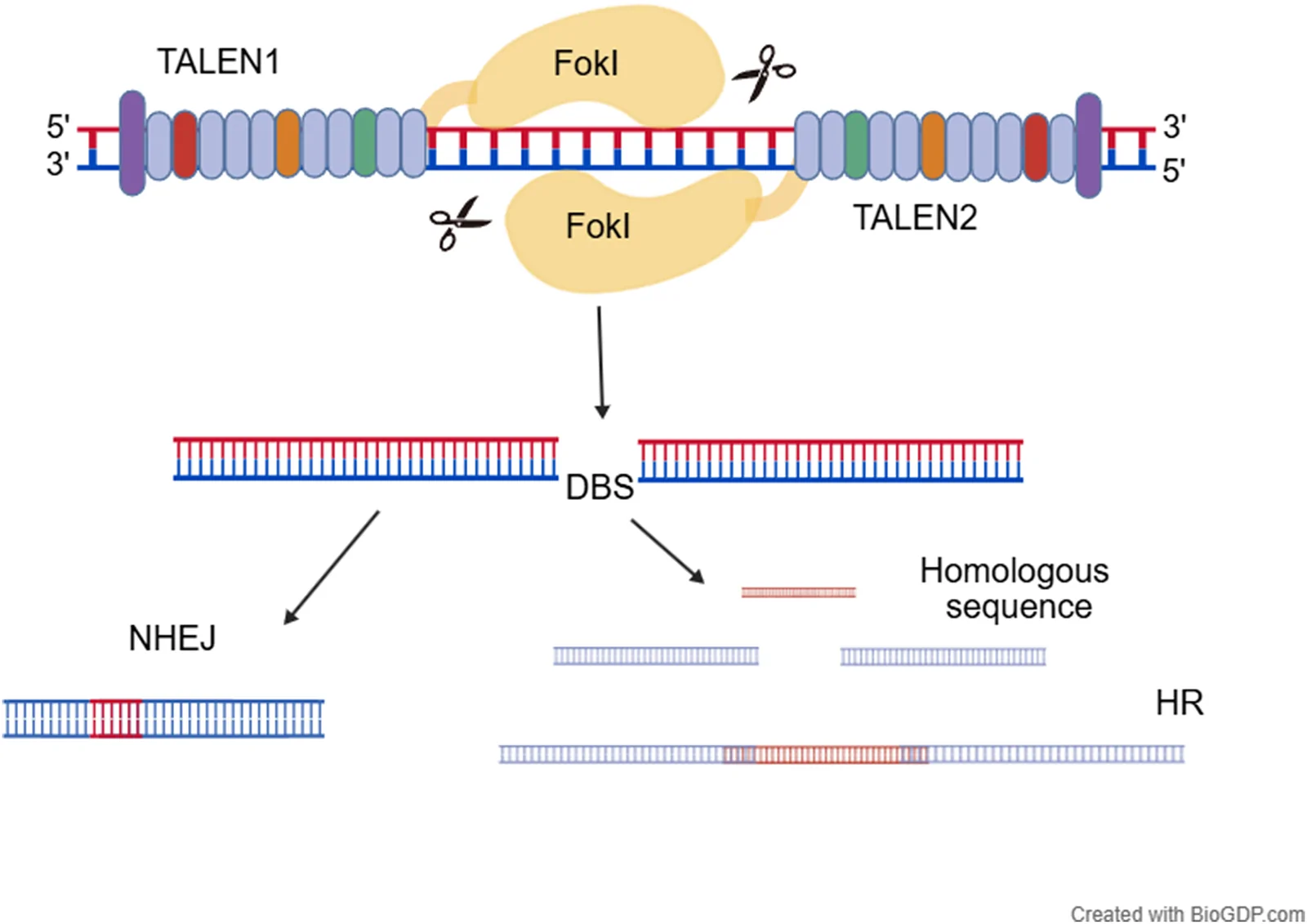
Schematic illustration of the mechanism of Transcription Activator-Like Effector Nuclease (TALEN)-mediated gene editing. The TALEN system consists of two engineered monomers (TALEN1 and TALEN2), each comprising a DNA-binding domain flanked by a FokI cleavage domain. These monomers bind specifically to the left and right target sequences flanking a central spacer region. Upon binding, the *Fok*I domains dimerize and introduce a site-specific double-strand break (DSB) at the target site. The resulting DSB is subsequently repaired by two distinct cellular pathways: (Left) Non-homologous end joining (NHEJ), which often leads to small insertions or deletions (indels) and gene disruption; and (Right) Homology-directed repair (HDR), which utilizes an exogenous homologous sequence as a template for precise gene correction or insertion. Abbreviations: TALEN, transcription activator-like effector nuclease; DSB, double-strand break; NHEJ, non-homologous end joining; HDR, homology-directed repair; HR, homologous recombination.

As a second-generation gene editing tool, TALENs possess distinct advantages over other gene editing technologies. One key benefit is their high specificity. Although, CRISPR-Cas9 is widely adopted, it relies on short guide RNAs (gRNAs) to mediate the targeting of the Cas9 nuclease; this RNA-mediated targeting mode is prone to off-target effects due to imperfect sequence complementarity between the gRNA and non-target genomic regions. In contrast, TALENs function through direct protein-DNA interactions; their DNA-binding domains can be engineered to precisely recognize specific target sequences, resulting in significantly higher specificity [19]. Another significant advantage over ZFNs and CRISPR-Cas9 is their broader range of targetable DNA sequences [20]. CRISPR-Cas9 requires a protospacer adjacent motif (PAM) near the target site, which restricts its applicability in certain genomic regions. By contrast, the modular nature of the TALE repeat domains allows TALENs to recognize and bind to almost any DNA sequence, regardless of the presence of a PAM sequence, offering greater flexibility in genomic targeting [8].

TALENs exhibit broad applicability across diverse plant species, whereas the efficiency of CRISPR-Cas9 is often more variable, primarily due to differences in stable transformation efficiency and the expression level of the editing components across species [21]. Heritable targeted mutations mediated by transcription activator-like effector nucleases (TALENs) were first established in *Arabidopsis thaliana* using the *Agrobacterium*-mediated floral dip method, by designing a pair of TALENs targeting two highly homologous paralogs within the *GLL22* tandem gene cluster, successfully induced a 4.4-kilobase (kb) deletion in this locus. These results demonstrate that TALENs, like ZFNs, can mediate large-scale chromosomal structural rearrangements, providing a powerful tool for the functional dissection of plant gene families [22]. Researchers utilized highly active promoters in shoot apical meristem (SAM) stem cells (such as *At1g76110*) to drive TALEN expression, resolving the core issue of low germline transmission efficiency in early TALEN technology in plants, and established a highly efficient and stable *Arabidopsis* TALEN editing system, and whole-genome resequencing failed to detect any true off-target mutations, demonstrating that TALENs is a highly precise plant genome editing tool [23]. Previous studies have demonstrated genetically homozygous C-to-T single-base editing in the mitochondrial genome of *Arabidopsis thaliana*, allowing for the generation of stable homozygous mutants as early as the T1 generation, this approach enables precise modification of mitochondrial genes underlying agronomic traits, including the creation and restoration of cytoplasmic male sterility (CMS) lines [24]. TALEN-mediated targeted mutagenesis of endogenous genes has been demonstrated in *Brassica oleracea* (cabbage), a member of the Brassicaceae family, resulting in the successful generation of cabbage mutants with loss-of-function mutations in the *FRI* gene. These mutants displayed a distinct early-flowering phenotype, indicating that TALEN technology can be effectively applied to non-model crops including cabbage [25]. MitoTALENs were used to achieve targeted modification of the oilseed rape mitochondrial genome in the Kosena-type cytoplasmic male sterility (CMS) line SW18 as the experimental model and successfully restoring male fertility in the edited plants [26]. As a second-generation gene editing technology, TALENs possess exceptionally high targeting specificity and minimal off-target effects, with negligible restrictions on target selection, making them a mature tool for editing the plant mitochondrial genome. However, several challenges remain, such as high costs associated with vector construction and cumbersome procedures; low editing efficiency in certain crops or genomic regions; and the difficulty of high-throughput screening of edited plants [17].

#### 2.1.3 CRISPR-Cas technology

The CRISPR-Cas system, which consists of CRISPR repeat-spacer arrays and Cas proteins, is prevalently found in bacteria and archaea. This system functions as an RNA-mediated adaptive immune mechanism defending against phages and other foreign genetic elements by cleaving the nucleic acid genomes of invading pathogens [9]. The CRISPR-Cas system is divided into two major categories. In the first category, the effector modules are multi-subunit complexes that rely on repeat-associated mysterious proteins (RAMPs) to form the core scaffold; common examples include Cas5, Cas7, Cas8 and Cas10. The second type of effector consists of a single large protein (monomeric effector), exhibiting a simpler and more unified CRISPR-Cas locus architecture. These include the widely used genome-editing enzyme Cas9, the predicted effector protein Cas12a (formerly known as Cpf1). The second type of CRISPR-Cas system is the primary source of gene-editing technologies (such as Cas9, Cas12a, and Cas13a); their single-protein effector nature renders them more amenable to engineering [27].

At present, the most widely used CRISPR-Cas systems are the Cas9 and Cas12a (also known as Cpf1) complexes; both are single-effector proteins with nucleic acid cleavage functions [6]. CRISPR-Cas9 technology originated from the type II CRISPR-Cas system; as shown in Fig. 3, Cas9, as a CRISPR-associated endonuclease, utilizes the tracrRNA: crRNA duplex to form complementary base pairs with the target DNA sequence, thereby introducing double-strand breaks (DSBs) at specific sites, the tracrRNA: crRNA duplex is designed as a single-guide RNA (sgRNA), retaining two key features: the 5′-end sequence determines the DNA target site via Watson-Crick base pairing, and the 3′-end double-stranded RNA structure binds to Cas9 [28]. Since only the guide sequence of sgRNA needs to be modified, the CRISPR–Cas9 system can be readily reprogrammed to target virtually any DNA locus. Its advantages of ease of use, a unique cleavage mechanism, and strong multi-target editing capabilities make it a low-cost, user-friendly and highly efficient editing tool [29]. Cas12a (Cpf1) is a single-RNA-guided nuclease that does not require tracrRNA; it recognizes T-rich protospacer adjacent motifs (PAMs) and cleaves target DNA by generating staggered double-strand breaks [30]. Currently, both systems can achieve precise targeting, editing and regulation of multiple genomic loci in plants, and significant progress has been made in plant genome editing research.

**Fig. 3:**
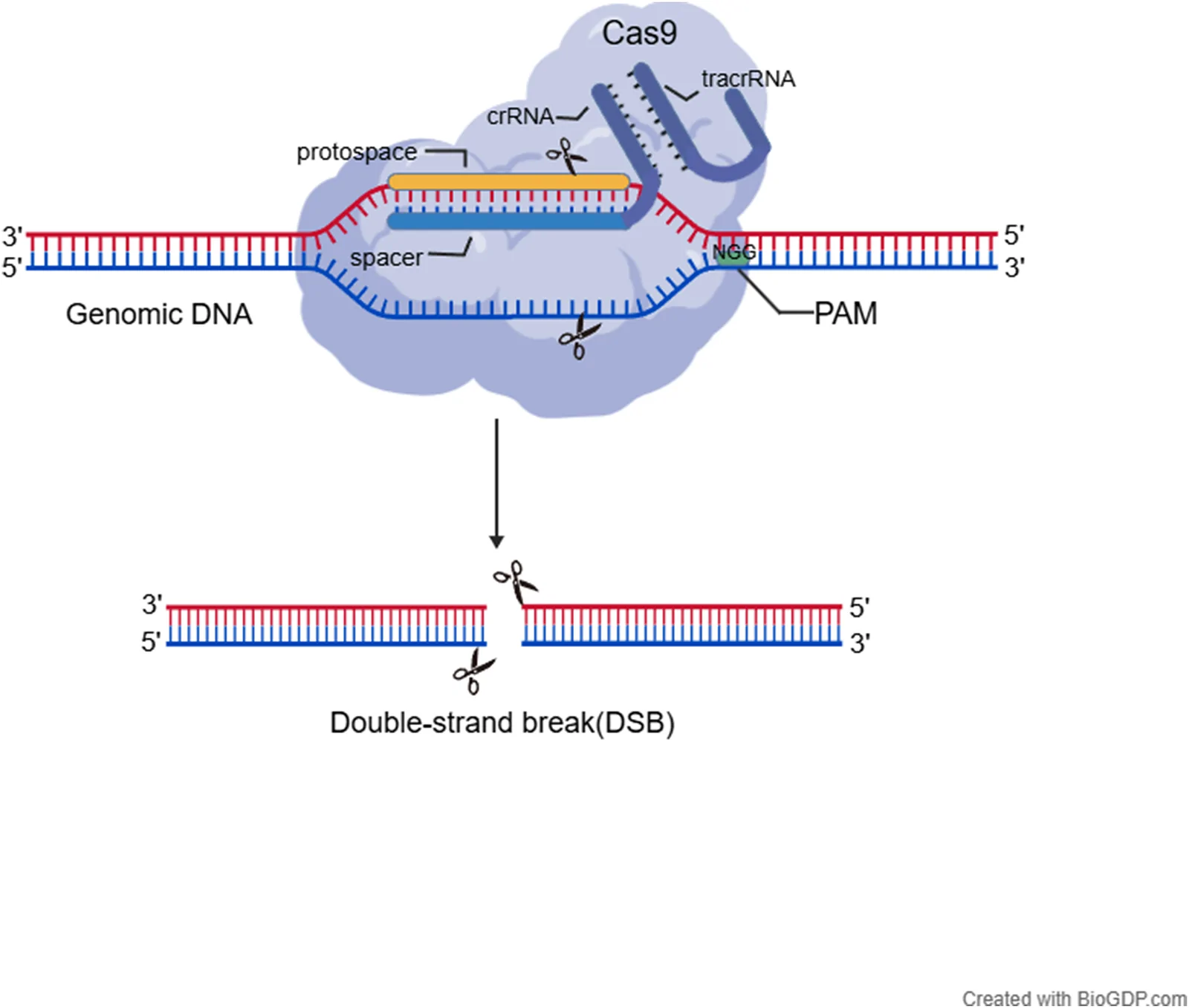
Schematic illustration of the CRISPR-Cas9-mediated site-specific DNA double-strand break (DSB). The Cas9 nuclease forms a ribonucleoprotein complex with a guide RNA comprising CRISPR RNA (crRNA) and trans-activating CRISPR RNA (tracrRNA). This complex recognizes and binds to the target genomic DNA through complementary base pairing between the crRNA spacer sequence and the DNA protospacer sequence, which strictly requires the presence of an adjacent protospacer adjacent motif (PAM) (e.g., NGG) for target recognition. Following binding and R-loop formation, the Cas9 nuclease domains cleave both strands of the DNA at specific sites within the protospacer (indicated by scissors), resulting in a site-specific double-strand break (DSB). Abbreviations: DSB, double-strand break; PAM, protospacer adjacent motif; crRNA, CRISPR RNA; tracrRNA, trans-activating CRISPR RNA.

An efficient CRISPR-Cas9 genome editing system was established in *Arabidopsis thaliana* and *Nicotiana benthamiana*, demonstrating the feasibility of this technology in plants and paving the way for the widespread application of genome editing in plant biology [31]. In 2015, a stable CRISPR editing system for *Brassica oleracea* was first established, obtaining stably heritable targeted mutants [32]. CRISPR-Cas9-mediated simultaneous knockout of multiple homologous genes has been achieved in allopolyploid oilseed rape, overcoming a core challenge in genome editing of polyploid crops. A complete CRISPR-Cas9 editing workflow was established for oilseed rape, providing a valuable reference for gene editing in other polyploid plant species [33]. The editing efficiency, mutation patterns, genetic characteristics and specificity of CRISPR-Cas9 in rapeseed were systematically evaluated at the whole-genome level, establishing a technical framework for simultaneous multi-gene editing in this species. CRISPR-Cas9 showed high editing efficiency in allopolyploid rapeseed: the average editing efficiency for single genes was 65.3%, while the efficiency for simultaneous editing of multiple genes reached 86.2%. By designing sgRNAs targeting conserved sequences within gene families, homozygous mutants for multiple genes could be obtained in a single generation, addressing the challenge of gene redundancy in polyploid crops [34]. Research team successfully created rapeseed germplasm with high resistance to bacterial black spot and excellent agronomic traits using CRISPR-Cas9 technology, providing a novel strategy for disease-resistant rapeseed breeding. The *prt6* quadruple mutant they created is currently the first rapeseed germplasm, both domestically and internationally, to achieve high resistance to bacterial black spot and excellent agronomic traits through gene editing, and can be directly utilized in breeding practice [35]. Cabbage is a typical sporophytic self-incompatible crop. Propagation of its inbred lines requires manual bud pollination, which is labor-intensive, costly, and prone to variety degeneration. CRISPR-Cas9-mediated genome editing has been employed to simultaneously overcome self-incompatibility in cabbage and generate economically viable nuclear male-sterile lines, which accelerates the cabbage breeding process and provides a foundation for technological innovation in hybrid seed production of Brassicaceae crops [36]. A stable CRISPR-Cas9 gene editing system has been established in radish, which was used to verify the molecular mechanism underlying leaf trichome formation in this species. The single-gene editing efficiency reached 75%, while the simultaneous dual-gene editing efficiency was 62.5%. Hairless radish germplasm was generated via gene editing, and homozygous mutants free of transgenic components were successfully isolated, providing valuable breeding material for the development of new leaf radish varieties with improved flavor [37]. A single-gene editing efficiency of 75% and a dual-gene editing efficiency of 62.5% were achieved. The LbCas12a system has been applied to *Brassica oleracea*, enabling the generation of T-DNA-free homozygous mutants in the T1 generation. All induced mutations exhibited stable heritability, providing an efficient tool for gene editing in dicotyledonous crops [38]. As a typical allopolyploid species, *Brassica juncea* possesses a highly complex genome characterized by multiple homologous gene copies, extensive repetitive sequences, and high heterozygosity. Consequently, conventional breeding and transgenic approaches are often inefficient for simultaneously manipulating multiple homologous genes. However, the application of CRISPR/Cas9 technology enables concurrent targeting of multiple loci, thereby facilitating rapid generation of loss-of-function mutants and efficient improvement of agronomic traits [39].

CRISPR-Cas systems are currently extensively applied in genome editing of Brassicaceae crops and have emerged as the predominant platform, yet they still encounter multiple technical challenges. For example, this system still produces persistent off-target effects that cannot be completely eliminated, PAM sequences limit the target selection range, the efficiency of precise gene knock-in mediated by homology-directed repair (HDR) is much lower than that of non-homologous end joining, the editing efficiency varies significantly between different species and genomic regions, multiplex gene editing and large-fragment genome manipulation are difficult, and the genetic transformation systems of some crops are imperfect.

#### 2.1.4 Base editing technology

Base editing technology was first proposed in 2016. It overcomes the drawbacks of conventional CRISPR-Cas9, which depends on DNA double-strand breaks (DSBs) and exogenous donor templates. This technology enables accurate single-base substitutions and has paved the way for further progress in genome editing. As shown in Fig. 4, the core principle of base editing technology involves three core steps: 1. Specific targeting on a modified CRISPR-Cas9 system for specific localization; 2. Base chemical conversion through fused cytidine deaminase: deamination reaction of cytosine (C) → uracil (U); 3. The transient U:G mismatch into a stable T:A pairing by exploiting the cellular DNA repair mechanism [40]. Base editing technology represents a vital expansion of the CRISPR system, with the core advantage of directly achieving precise single-base conversion without DNA double-strand breaks (DSBs) or donor templates. It is divided into three categories: 1. Cytosine base editors (CBEs); 2. Adenine base editors (ABEs); 3. RNA base editors (RBEs, represented by the REPAIR system) [41].

**Fig. 4:**
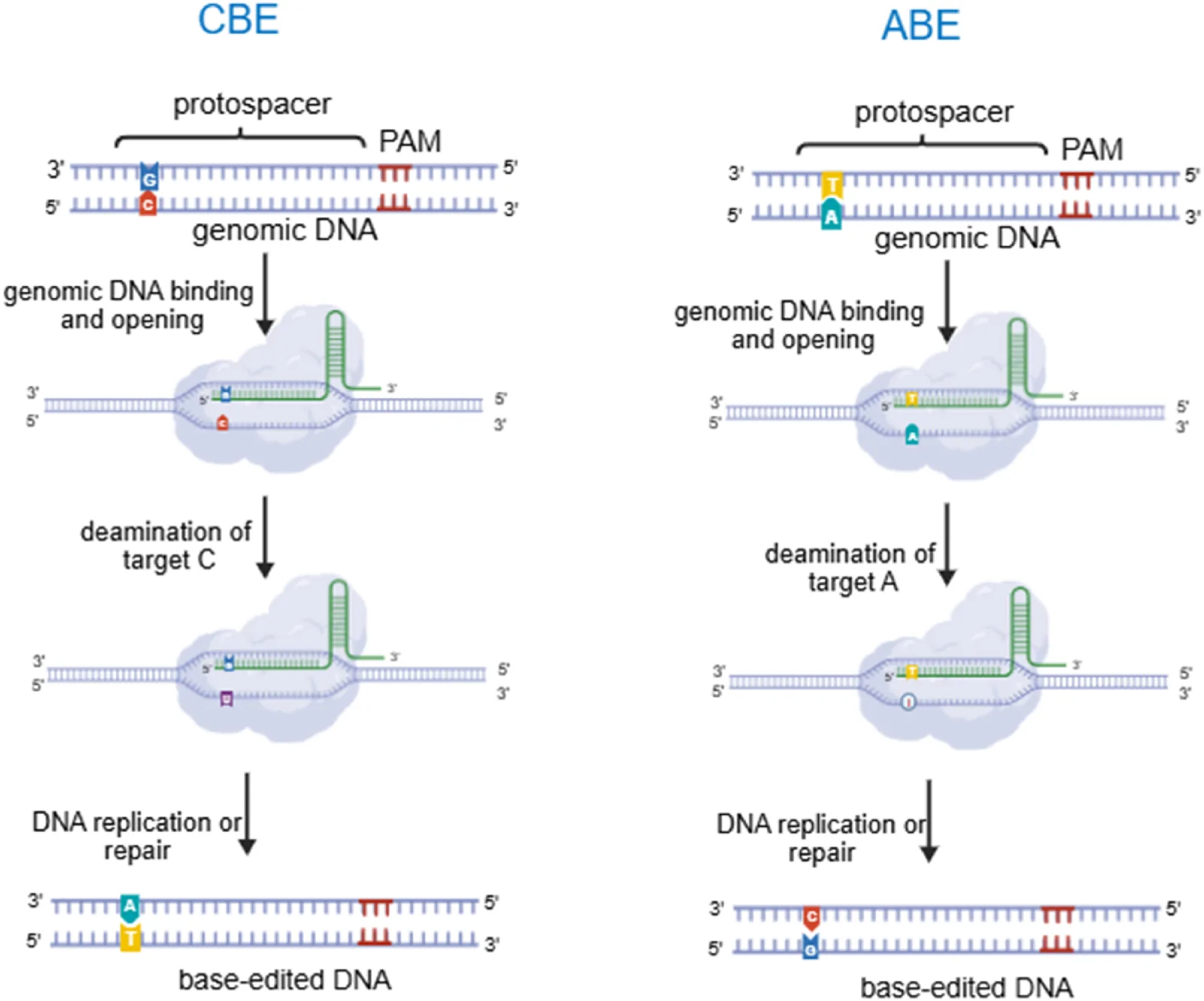
Schematic illustration of the mechanisms of cytosine base editor (CBE) and adenine base editor (ABE) mediated base editing. The process initiates with the specific binding of the base editor (gray structure) to the target genomic DNA at the protospacer and protospacer adjacent motif (PAM) sequences, accompanied by the formation of an R-loop. (Left panel) In the CBE pathway, the target cytosine (C) undergoes deamination to generate uracil (U). Subsequent DNA replication or repair processes result in a stable C-to-T transition, yielding the final base-edited DNA. (Right panel) In the ABE pathway, the target adenine (A) undergoes deamination to generate inosine (I). Due to the base-pairing properties of inosine, subsequent DNA replication or repair results in a stable A-to-G transition. Abbreviations: CBE, cytosine base editor; ABE, adenine base editor; PAM, protospacer adjacent motif.

Researchers developed an efficient and heritable C-to-T base editing system in *Arabidopsis thaliana*. Using this system, they generated herbicide-resistant point mutations at the *ALS* locus. This result demonstrated, for the first time, that CBE3 functions effectively in this dicot model, and further revealed that the PAM region is readily editable, thereby providing a valuable tool for precise breeding of dicot crops. [42]. Building on previous advances, four distinct cytosine base editor (CBE) variants were evaluated for their performance in allotetraploid rapeseed, and a cytosine base editor designated *BnA3A1-PBE* was developed, which exhibited superior editing performance in rapeseed. It achieved an average editing efficiency of 29.8% with a peak value of 50.5%, representing approximately 4–16-fold improvement over the conventional BE3 system. Using this optimized editor, novel rapeseed germplasms with dwarf plant architecture and herbicide tolerance were generated. This work addresses an important technical gap in high-efficiency CBE application for rapeseed [43]. An efficient A3A-PBE cytosine base editing system has been systematically established in allotetraploid rapeseed. Editing efficiencies exceeded 20% for all three target genes, with a maximum efficiency of 31.25%, representing a 13–17-fold improvement over the traditional BE3 system. The editing characteristics of A3A-PBE in rapeseed were clarified: this system can simultaneously edit multiple homologous genes and efficiently generate homozygous and biallelic mutants, leading to the successful creation of novel rapeseed germplasms with important breeding value [44]. A cytosine base editor recognizing NG PAM was employed to achieve precise C→T conversion at the P9 site of the *BnaCLV3* gene in rapeseed. Two mutation types, P9L and P9F, were obtained, which conferred the favorable trait of multilocular siliques while maintaining normal growth and development characteristics [45].

An efficient adenine base editing (ABE) system has been established in *Arabidopsis thaliana* and rapeseed. Late-flowering mutants were generated by editing the *FT* gene, and albino mutants were obtained by targeting the *PDS3* splice site. This work demonstrated that ABE can induce heritable phenotypic changes in plants, establishing a foundation for A-to-G single-base editing in plant species [46]. Stable adenine base editing has been achieved in allotetraploid rapeseed. This system complements cytosine base editor (CBE) technology to enable all four possible single-base transitions, expanding the base editing toolkit for rapeseed [47].

In contrast, RNA base editing, with the REPAIR system as a typical example, has not been applied in Brassicaceae for the time being. Base editing technology can achieve precise single-base modification at specific genomic sites without inducing DNA double-strand breaks, and has the advantages of high editing efficiency, high product purity, and relatively low off-target effects. However, it still has several limitations, including a narrow range of base conversion types, non-specific off-target editing, sequence context preferences, incomplete mitigation of off-target risks in certain systems, and suboptimal delivery efficiency.

## 3 Current status of gene editing research in Brassicaceae crops

### 3.1 Advances in genome editing for the improvement of important agronomic and quality traits

Bolting and flowering, plant architecture, and nutritional quality represent core target traits in the genetic improvement and production application of Brassicaceae crops. They respectively determine the environmental adaptability of growth duration, yield potential, and commercial nutritional value, and constitute key directions for cultivar breeding and germplasm innovation. Plant architecture directly affects radiation use efficiency and grain yield, thereby influencing oil yield, and is a key agronomic trait determining rapeseed yield. As shown in Fig. 5, numerous important regulatory genes underlying agronomic and quality traits have been identified in Brassicaceae via genome editing technology.

**Fig. 5:**
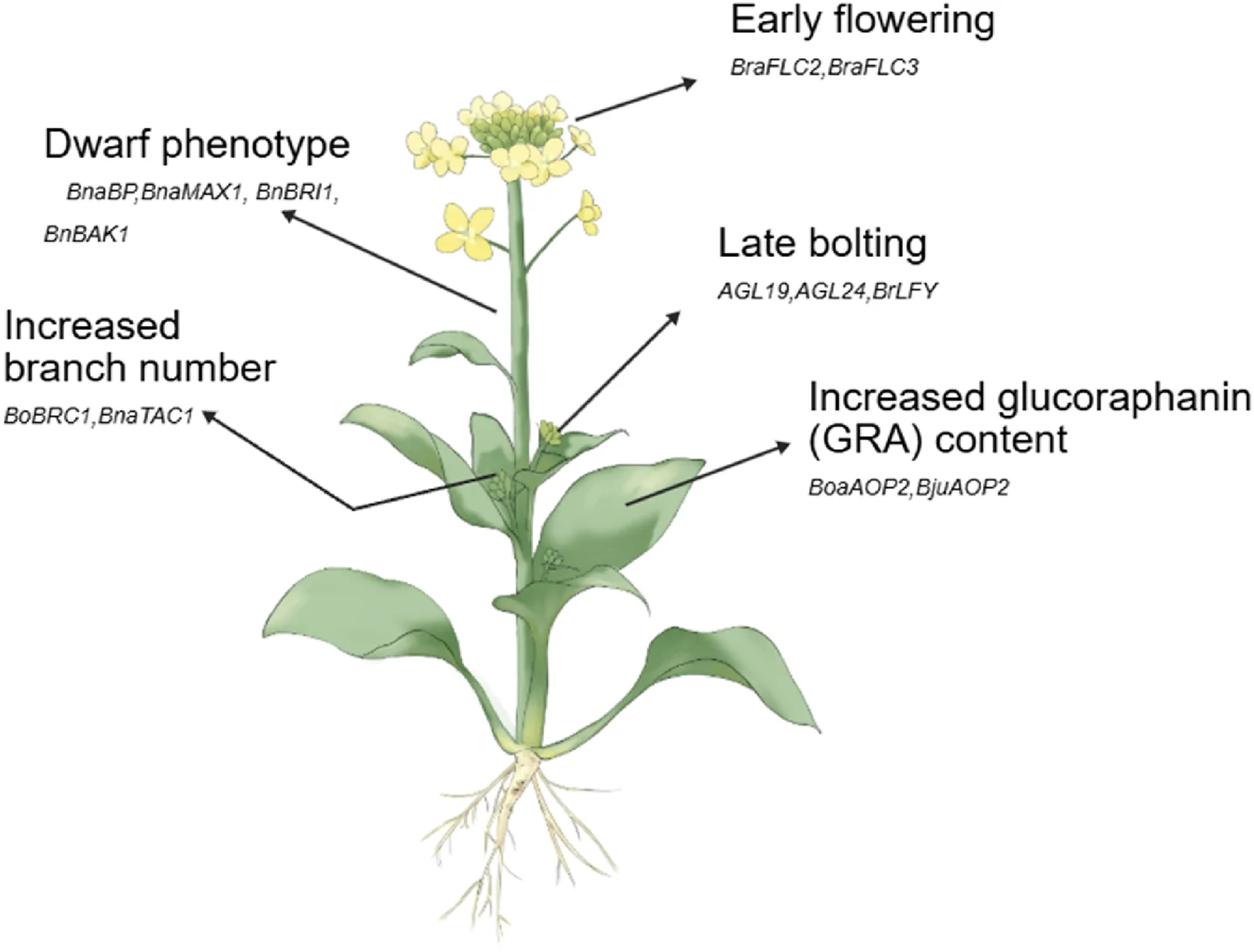
Key phenotypic regulatory genes identified via genome editing in Brassicaceae. *FLC2* and *FLC3* belong to the homologous gene family of FLOWERING LOCUS C (*FLC*) [48]. *AGL19* and *AGL24* belong to the AGAMOUS-like (*AGL*) subfamily of the MADS-box gene family [49]. *BrLFY*, *Brassica rapa* LEAFY [50]. *BoBRC1*,*Brassica oleracea* BRANCHED 1 [51]. *BnaMAX1*,*Brassica napus* MORE AXILLARY GROWTH 1 [52]. *BnaBP*, *Brassica napus* BREVIPEDICELLUS [53]. *BoaAOP2*,*Brassica oleracea* var. alboglabra ALKENYL HYDROXYALKYL PRODUCING 2 [54]. *BjuAOP2*,*Brassica juncea* ALKENYL HYDROXYALKYL PRODUCING 2 [55].

#### 3.1.1 Bolting and flowering

Bolting and flowering mark the key developmental shift from vegetative to reproductive growth in Brassicaceae. Most of these traits are quantitative traits controlled by multiple genes and regulated by multiple pathways integrating environmental signals and endogenous factors. The Brassicaceae family encompasses a wide range of crop types. For crops whose main edible parts are flower stems, flower buds, and siliques, timely and appropriate promotion of bolting and flowering often has important production value, such as flowering Chinese cabbage and purple flowering stalk. For crops whose main edible parts are modified roots, stems, leaves and other vegetative organs, effective prevention of premature bolting and flowering is the core premise to ensure their high and stable yield [56].

Flowering and bolting time are also key considerations in crop breeding programs. Therefore, the genetic and molecular regulatory mechanisms of bolting and flowering in Brassicaceae crops have always been a major research focus for researchers worldwide. A few research groups applied CRISPR-Cas9 to edit homologous copies of the flowering repressor gene *BraFLC* in Chinese cabbage (*Brassica rapa* ssp*. pekinensis*). This work generated early-flowering lines that no longer require vernalization. They clarified the functional redundancy of *BraFLC2* and *BraFLC3* and established an efficient Chinese cabbage genome editing platform [48]. Other studies used CRISPR-Cas9 technology to simultaneously knock out the *AGL19* and *AGL24* genes in Chinese cabbage, successfully creating new transgene-free late-bolting Chinese cabbage germplasm. Compared with wild-type plants, these mutants showed a 10–11 day delay in bolting, meanwhile, their vegetative and reproductive growth remained normal with no undesirable agronomic traits [49]. In addition, by using CRISPR-Cas9 technology targeting and editing the homologous gene of the flower meristem identity gene *BrLFY* in Chinese cabbage (*Brassica rapa* L. ssp*. pekinensis*), a transgene-free (T-DNA-free) late-bolting line was successfully created, addressing the problem of premature bolting and concomitant yield and quality decline of Chinese cabbage caused by early spring climate change [50]. Similar editing strategies were also applied to rapeseed. New transgene-free early-flowering germplasms were obtained, with flowering time advanced by around 8 days relative to wild types. This study improved our understanding of the rapeseed flowering regulatory network, and provided a new solution for the study of flowering mechanisms in Brassicaceae plants [57].

Bolting and flowering mark a vital developmental shift from vegetative to reproductive growth in Brassicaceae crops. This transition directly determines yield, quality and cultivation adaptability, and is the core research focus in crop breeding and germplasm innovation. Currently, gene editing has become the core tool for flowering regulation research and practical breeding applications in Brassicaceae.

#### 3.1.2 Plant architecture

Plant architecture, a core agronomic trait that determines yield for most Brassicaceae crops (primarily the genus *Brassica*), exerts profound impacts on crop yield, product quality, stress tolerance and mechanical harvesting suitability. It also influences planting density, light interception efficiency and overall field management costs. Rapeseed is an important oil crop in China, and the seed yield of rapeseed varieties determines the economic value of rapeseed. Plant architecture directly affects light energy utilization efficiency and seed yield. The ideal plant architecture (compact plant architecture + low branching position + strong lodging resistance + multiple branches) can increase the yield per plant of rapeseed by 20–30% and the population yield by 15–25% [58]. Other studies edited homologous copies of *BnaMAX1*, *BnBRI1*, *BnBAK1*and *BnaBP* in *Brassica napus* via CRISPR-Cas9; these genes are involved in strigolactone biosynthesis and brassinosteroid signaling, respectively, and their targeted knockout generated semi-dwarf and compact germplasms with reduced lodging susceptibility and improved mechanical harvest performance [52,53,[59], [60], [61]]. Furthermore, *BnaTAC1* exhibits preferential and high expression in lateral branches, with markedly lower expression levels in other tissues; CRISPR/Cas9-mediated knockout of *BnaTAC1* produced double mutants with significantly reduced branch angles, a trait that facilitates high-density planting and mechanical harvesting. These findings collectively demonstrate that CRISPR-Cas9-mediated manipulation of multiple genetic nodes can effectively optimize plant architecture in rapeseed [61]. In addition, the number of lateral branches of broccoli directly affects yield and cultivation purpose: sprouting broccoli requires more lateral branches to increase yield. Related research adopted CRISPR-Cas9 genome editing to characterize the function of *BoBRC1*, the results revealed that this gene restricts lateral branch growth by modulating its downstream targets [51].

#### 3.1.3 Nutritional quality

Brassicaceae crops are rich in bioactive substances such as glucosinolates, vitamins (vitamin C, folic acid, tocopherols and phylloquinone), carotenoids, phenolic compounds and various minerals, which are of great significance for diversifying human dietary patterns and maintaining human health [62]. By employing the CRISPR-Cas9 system, the upstream open reading frame (uORF) of *GGP*, which encodes the rate-limiting enzyme in the ascorbic acid (AsA) biosynthesis pathway, was edited in non-heading Chinese cabbage. The modification effectively disrupted the feedback inhibition of AsA synthesis, resulted in a substantial increase in endogenous AsA accumulation, and generated novel germplasm with elevated AsA levels. These elite materials provide core resources for the directional improvement of nutritional quality in non-heading Chinese cabbage [63]. Three homologous *BoaAOP2* genes were first identified and cloned from kale, targeted mutagenesis of these genes via CRISPR/Cas9 editing remodelled the metabolic flux of aliphatic glucosinolate (GSL) side-chain modification, thereby significantly promoting the accumulation of glucoraphanin (GRA), a key bioactive compound with anticancer properties. This edited germplasm can be directly applied as parental materials for the high-nutrition quality breeding of kale, offering crucial germplasm support for the development of functional vegetables enriched with anticancer components [54]. CRISPR-Cas9 multiplex editing technology was used to target five *BjuAOP2* homologous genes in mustard (*Brassica juncea*). This approach successfully blocked the synthesis of anti-nutritional alkenyl glucosinolates, achieved ultra-high accumulation of glucoraphanin (GRA), and maintained stable agronomic traits of the edited plants [55]. The hydrolysis products of aliphatic glucosinolates (A-GSL), such as sulforaphane, have health benefits, but excessive accumulation of A-GSL in some Brassicaceae crops may affect quality. Relevant research teams targeted and knocked out functional copies of the *MYB28* gene family in *Brassica oleracea* through CRISPR-Cas9 technology, inversely proving that the *MYB28* gene is a key regulator of aliphatic glucosinolate (A-GSL) synthesis, providing valuable insights for the targeted improvement of nutritional quality of Brassicaceae crops [64].

The cuticular wax on the surface of broccoli (mainly containing alkanes, alcohols, fatty acids, etc.), is a barrier for plants to resist biotic and abiotic stresses and an important quality trait. Related research adopted CRISPR-Cas9 to edit the *BoGL5* gene in broccoli (*Brassica oleracea* var*. italica*), this work clarified the vital function of *BoGL5* in cuticular wax biosynthesis and generated new stably inherited glossy green broccoli lines [65].

Rapeseed is an important oil crop globally, and its oil quality is mainly determined by fatty acid composition. Oleic acid (18:1) content has become a key target trait in rapeseed breeding due to its oxidative stability, extended shelf life, and cholesterol-lowering health benefits. Accordingly, researchers used CRISPR-Cas9 to edit functional copies of the *BnaFAD2* gene family in *Brassica napus*, thereby improving the fatty acid profile, increasing oleic acid content, and providing valuable germplasm for breeding improved oil quality [66].

Mustard is a nutritious leafy vegetable abundant in vitamins, minerals and phenolic compounds; however, its strong pungent taste when eaten fresh limits fresh consumption. The reason for this pungent taste is that when plant tissue is damaged by chewing, myrosinase comes into contact with glucosinolate substrates such as sinigrin, and undergoes a hydrolysis reaction to form highly irritating isothiocyanates. Related studies targeted and edited 17 functional class I myrosinase genes in allotetraploid mustard (*Brassica juncea*) through CRISPR-Cas12a technology, successfully blocking the hydrolysis reaction between myrosinase and glucosinolates, achieving a stable reduction in the pungent taste of fresh mustard leaves. The created non-pungent mustard germplasm can be eaten directly fresh, broadening the consumption scenarios of mustard [67].

Gene editing technology has been widely used in Brassicaceae vegetables and oilseed crops. Precise modulation of key genes involved in nutrient and flavor metabolism enables targeted enhancement of ascorbic acid, beneficial glucosinolates and oleic acid. Multiple high-quality germplasms ideal for fresh consumption have been developed, supporting directional nutritional breeding of Brassicaceae crops.

### 3.2 Advances in genome editing for the improvement of important stress tolerance traits

Abiotic stresses (e.g., temperature, salinity, alkalinity, and heavy metals) and biotic stresses (e.g., diseases and insect herbivory) are core adverse factors limiting the growth, development, yield formation, and quality improvement of Brassicaceae crops. They directly affect the regional cultivation adaptability and field production stability of crops, and represent a key research direction for genetic improvement of crop stress tolerance. As shown in Table 1, numerous important stress-associated regulatory genes have been identified in Brassicaceae via genome editing technology.

**Table 1:** Key stress-related regulatory genes identified via genome editing in Brassicaceae.

Stresses	Gene	Gene-edited phenotypes	Protein category	Mechanism	Reference
Temperature stress	*CBF*	Reduced freezing tolerance in *CBF*-edited lines relative to the wild type	Transcription factor	Upon perception of cold signals, plants rapidly induce CBF transcription. *CBF* proteins translocate into the nucleus, bind to CRT/DRE elements in the promoters of downstream *COR* genes, and transcriptionally activate a suite of freezing tolerance-related physiological processes.	Zhao et al., 2016 [65]
	*USP1*	*USP1*edited lines completely lost heat acclimation-induced tolerance and exhibited more severe growth inhibition.	Cysteine protease of peptidase family C19	*USP1* encodes a molecular chaperone that binds and stabilizes the master heat stress transcription factor *HSFA2*, facilitating H3K4me3 epigenetic modification at thermomemory gene loci to regulate plant thermopriming.	Manickam et al., 2026 [68]
Salt stress	*AITR*	Under salt stress, *AITR*-edited lines exhibited less severe growth inhibition, normal flowering and seed set, and significantly reduced yield loss relative to wild type (WT).	Transcription factor	As a transcriptional repressor, *AITR* forms a negative feedback loop by regulating the expression of core components in the ABA signaling pathway.	Chen et al., 2021 [69]
	*AtWRKY66*	Under salt (NaCl) and abscisic acid (ABA) treatments, *AtWRKY66*-edited lines exhibited suppressed growth and aggravated cell membrane damage.	Transcription factor	*AtWRKY66* is strongly induced by salt stress and ABA, and functions through the ABA-mediated signaling pathway.	Zhang et al., 2023 [70]
	*BnaMPK6*	*BnaMPK6*-edited lines displayed an obvious salt-sensitive phenotype with suppressed growth and development.	CMGC kinase superfamily, MAPK subfamily	*BnaMPK6* participates in the plant SOS (Salt Overly Sensitive) salt-tolerance pathway. Activated by salt stress, it binds and activates plasma membrane Na^+^/H^+^ antiporter SOS1 to promote cellular Na^+^ efflux and maintain intracellular ion homeostasis.	Zhang et al., 2023 [71]
Drought stress	*BnNAC038*	*BnNAC038*-edited lines exhibited significantly enhanced drought tolerance at the seedling stage, with yield loss being substantially lower than that of the wild-type plants.	Transcription factor	Drought/ABA signaling suppresses *BnNAC038* expression and alleviates its inhibition of downstream targets, which simultaneously promotes stomatal closure to curtail water loss and de-represses photosynthesis- and sugar-metabolism-related genes, thereby sustaining higher photosynthetic activity and growth potential under drought and resulting in substantially lower yield penalties.	Chen et al., 2025 [72]
	*BnPUB12*	*BnPUB12*-edited lines showed severe leaf wilting, curling, and chlorosis.	U-box (PUB) E3 ubiquitin ligase family	*BnPUB12* belongs to the U-box E3 ubiquitin ligase family and positively regulates ABA signaling, thereby promoting stomatal closure and the expression of antioxidant genes.	Zhou et al., 2026 [73]
	*BnaABI4*	*BnaABI4*-edited lines exhibited significantly increased stomatal density and transpirational water loss, leading to reduced drought tolerance.	Transcription factor	*BnaABI4* is a core regulatory factor of the abscisic acid (ABA) signaling pathway and is involved in seed germination, seedling growth, stomatal development, and stress responses.	Yang et al., 2026 [74]
Cadmium (Cd) stress	*AtCUP1*	Under cadmium stress, *CUP1* knockout mutants exhibited prominent Cd tolerance and low Cd accumulation phenotypes.	MFS transmembra-ne transporters	The protein encoded by *AtCUP1* localizes to the plasma membrane and functions as a Cd^2+^ channel, directly mediating cadmium uptake from soil by plant roots.	Yao et al., 2022 [75]
	*WAKL4*	Under cadmium (Cd) stress, *WAKL4* knockout mutants displayed obvious Cd-sensitive phenotypes with suppressed growth and development.	WAK/WAKL subfamily of the RLK superfamily	*WAKL4* acts as a positive regulator of cadmium tolerance via the core *WAKL4*-*NRAMP1* module.	Yuan et al., 2024 [76]
Pathogen stress	*BoBPM6*	After inoculation with three major diseases, including fusarium wilt, black rot and clubroot, knockout mutants exhibited varying degrees of enhanced disease resistance.	Substrate adaptor proteins of CUL3-type E3 ubiquitin ligases	*BoBPM6* functions as a susceptibility gene and negatively regulates plant immunity.	Zhang et al., 2025 [77]
	*BnaA07.SUC2*	Upon infection by *Plasmodiophora brassicae*, *BnaA07.SUC2* knockout mutants displayed a markedly enhanced disease-resistant phenotype.	Major Facilitator Superfamily (MFS) transmembrane transporters	The molecular mechanism by which *BnaA07.SUC2*, a susceptible gene, mediates clubroot disease development centers on the JA-*BnaA05.MYC2-BnaA07.SUC2* regulatory module.	Liu et al., 2026 [78]
	*eIF(iso)4E*	After inoculation with turnip mosaic virus (TuMV-GFP), *eIF(iso)4E* knockout mutants exhibited a significantly enhanced disease resistance phenotype.	RNA-binding proteins/translation initiation factors	Turnip mosaic virus (TuMV) genomic RNA lacks a 5′ cap structure. Its translation initiation relies on specific interaction between viral VPg and host *eIF(iso)4E*, leading to plant susceptibility.	Pyott et al., 2016 [79]
Insect herbivory stress	*bHLH19* *bHLH20*	Upon herbivory by *Spodoptera frugiperda* (fall armyworm) and *S. exigua* (beet armyworm), *bhlh19/20* double mutants exhibited markedly enhanced insect resistance.	Transcription factor	*bHLH19/20* repress the transcriptional activation activity of the *MYC*-*MYB* complex, downregulate the expression of downstream defense genes (e.g., glucosinolate biosynthetic genes), and thereby attenuate plant defense responses.	Pang et al., 2024 [80]
	*BnaFAH*	Under specific light conditions, *BnaFAH* mutant lines exhibited a phenotype with markedly enhanced insect resistance.	Metal-dependent hydrolase, belonging to the FAH superfamily	Loss of *BnaFAH* triggers reactive oxygen species (ROS) accumulation under short-day conditions, which further activates jasmonic acid (JA) biosynthesis and signaling pathways to ultimately boost plant resistance against diamondback moths.	Zhi et al., 2026 [81]

Note: *CBF* (C-repeat Binding Factor) proteins are also designated as *DREB1*. *USP1*,Ubiquitin Specific Peptidase 1. *AITR*,ABA-induced transcription repressors. *AtWRKY66*,*Arabidopsis thaliana* WRKY DNA-binding protein 66. *BnaMPK6*,*Brassica napus* Mitogen-Activated Protein Kinase 6.*BnNAC038*,*Brassica napus* NAC Transcription Factor 038.*BnPUB12*,*Brassica napus* U-Box E3 Ubiquitin Ligase 12.*BnaABI4*,*Brassica napus* ABA INSENSITIVE 4. *AtCUP1*,*Arabidopsis thaliana* Cadmium Uptake-related Protein 1. *WAKL4*,Wall-associated kinase-like 4. *BoBPM6*,*Brassica oleracea* BTB-POZ and MATH domain protein 6. *BnaA07.SUC2*,*Brassica napus* A07 Sucrose-proton symporter 2. *eIF(iso)4E*,Eukaryotic translation initiation factor isoform 4E. *bHLH19/20*,basic helix-loop-helix transcription factor 19/20. *BnaFAH*, *Brassica napus* Fumarylacetoacetate Hydrolase.

#### 3.2.1 Temperature stress

Temperature stress (high temperature/low temperature/freezing injury) is one of the most destructive abiotic stresses in the production of Brassicaceae crops, directly affecting yield, quality and stress resistance. Pioneering genome editing research generated homozygous triple deletion mutants of *CBF1/2/3* in *Arabidopsis thaliana*. These results confirmed that *CBF* genes are core factors for cold acclimation in *Arabidopsis thaliana*, ending the 20-year controversy over *CBF* function in the field of plant cold acclimation and establishing the core position of the *CBF* pathway in plant freezing tolerance [68]. High temperature and other environmental stresses can induce endoplasmic reticulum (ER) stress (accumulation of unfolded proteins), leading to growth inhibition, leaf yellowing and wilting, and seriously affecting yield and quality. Follow-up studies targeted and edited the *bZIP* domain of the *HY5* gene in Chinese cabbage (*Brassica rapa*) through CRISPR-Cas9 technology, clarifying its negative role in ER stress response [82]. Additional CRISPR editing assays knocked out the *BoNAP* gene in *Brassica oleracea* var*.italica*, creating new germplasm that is tolerant to both 42 °C high temperature and −9 °C freezing stress. It significantly improved the adaptability of broccoli to extreme temperatures by inhibiting leaf senescence induced by temperature stress, maintaining photosystem II activity and cell membrane integrity [69]. Other tests constructed *Arabidopsis thaliana USP1* homozygous loss-of-function mutants using CRISPR-Cas9, and found that *USP1* specifically regulates plant thermopriming (acquired thermotolerance) without affecting basal thermotolerance and normal growth and development [70]. At present, there is still insufficient research on the tissue-specific and developmental stage-specific regulatory mechanisms of temperature response in Brassicaceae crops. Research on gene editing technology in the field of temperature stress in Brassicaceae crops is particularly scarce, and the genetic basis of heat tolerance in important economic crops such as rapeseed (*Brassica napus*) remains poorly characterized at the genome-wide level.

#### 3.2.2 Salinity-alkali stress

Salt stress seriously restricts the yield and quality of food crops by destroying photosynthetic metabolism, ion balance and redox homeostasis. Salinity-alkali stress causes impaired crop water uptake through osmotic stress, and ion toxicity (excessive accumulation of Na^+^ and Cl^−^) disrupts cellular ion homeostasis, thereby inhibiting seed germination and plant growth and development, damaging the photosynthetic apparatus and antioxidant system, triggering oxidative stress, and ultimately leading to reduced crop yield and deteriorated quality. The degree of damage varies with crop species, growth stage and saline-alkali concentration [83]. Genome editing assays using the model plant *Arabidopsis thaliana* were employed to knockout the entire *AITR* gene family. The resulting mutants exhibited markedly improved drought and salt tolerance with no penalty on normal plant growth. This strategy can enhance plant stress resistance without compromising normal development, revealing the functional role of feedback regulators in plant salt stress response [71]. CRISPR-Cas9 technology was employed for follow-up functional characterization to clarify the key role of *AtWRKY66* as a positive regulator of the abscisic acid (ABA) signaling pathway in plant salt stress responses, providing a research direction for molecular breeding of saline-alkali tolerant crops [84]. Other CRISPR-mediated mutagenesis knocked out the NAC transcription factor gene *BoNAP* in broccoli (*Brassica oleracea* var*. italica*). Multiple abiotic stress assays were conducted on mutant lines covering saline-alkali exposure, drought, extreme temperature, intense UV radiation and dark treatment. Loss of *BoNAP* greatly boosted broccoli salt tolerance while simultaneously strengthening resistance to drought and thermal stress [69]. CRISPR-Cas9 technology was employed for further mutagenesis studies to generate complete loss-of-function mutants of *AtJAR1*. This work uncovered a mechanism by which the jasmonic acid (JA) signaling pathway regulates potassium ion uptake and K^+^/Na^+^ homeostasis through cross-talk with abscisic acid (ABA), defining its role in plant salt stress responses. Knockout of *AtJAR1* attenuates the inhibitory effects of salt stress on seed germination and root growth, thereby enhancing plant salt tolerance [85]. Additional genome editing work targeted and edited four homologous copies of the *BnaMPK6* gene in *Brassica napus* through CRISPR-Cas9 technology, systematically verifying its regulatory function in salt tolerance. *BnaMPK6* positively improves the oxidative stress tolerance level of *Brassica napus*, reduces excessive reactive oxygen species (ROS) accumulation and cell membrane damage induced by salt stress, and maintains normal plant growth; loss-of-function mutations in this gene can lead to disrupted redox homeostasis and significantly increased plant salt sensitivity [75]. Related rapeseed editing studies knocked out the *BnaA2.HKT1* gene in rapeseed (*Brassica napus*) using CRISPR-Cas9, revealing the molecular mechanism by which the trace element boron regulates plant salt tolerance, they found that boron promotes xylem Na^+^ unloading by specifically upregulating the expression of the root stele Na^+^ transporter *BnaA2.HKT1*, thereby reducing Na^+^ translocation to aerial tissues, providing a novel strategy for crop improvement in saline-alkali soils [76]. Current research on the molecular mechanisms of salt tolerance in Brassicaceae crops remains limited to the identification of some salt stress-related genes, lacking systematic analysis of the underlying regulatory networks; targeted modification of specific genes via CRISPR-Cas9 technology will facilitate deeper elucidation of the salt tolerance regulatory network of Brassicaceae crops.

#### 3.2.3 Drought stress

*Brassica napus* is highly sensitive to drought stress at both the seedling and reproductive stages, with drought conditions leading to reduced photosynthetic efficiency, impaired vegetative growth, and substantial yield losses. In recent years, CRISPR/Cas9-mediated genome editing has been widely employed for functional characterization and genetic improvement of drought tolerance genes in this species. Among negative regulators, *BnNAC038* has been identified as a negative regulator of drought tolerance; loss-of-function mutants generated via CRISPR/Cas9 editing of its homologous copies exhibited a survival rate exceeding 80% after 13 days of drought treatment, compared with less than 20% in the wild type, accompanied by slower leaf water loss, smaller stomatal aperture, and significantly decreased stomatal conductance and transpiration rate [72]. In contrast, *BnPUB12* was confirmed as a positive regulator of drought tolerance, as its knockout mutants displayed pronounced leaf wilting, curling, and chlorosis under drought stress, further substantiating its protective role in drought responses [73]. Moreover, the function of *BnaABI4* was elucidated through CRISPR/Cas9-generated knockout mutants, which exhibited the most severe wilting phenotype and the poorest recovery upon rewatering, demonstrating that *BnaABI4* positively regulates drought tolerance while negatively modulating stomatal density, thereby expanding the conventional understanding of *ABI4* functions [74]. Concurrently, studies on *BnABI5* revealed that its knockout accelerates seed germination under drought conditions by modulating a complex gene regulatory network involving glutathione metabolism, ABA signaling, and glucosinolate biosynthesis, thereby effectively enhancing drought tolerance at the germination stage in rapeseed [86]. Current research on the molecular mechanisms of drought tolerance in Brassicaceae crops remains largely confined to functional characterization of individual transcription factors and signaling components, with limited systematic dissection of the underlying regulatory networks; CRISPR/Cas9-mediated genome editing will facilitate deeper elucidation of drought tolerance mechanisms and accelerate the breeding of drought-resistant varieties in Brassicaceae crops.

#### 3.2.4 Heavy metal stress

The development of heavy industry has driven socioeconomic progress, but has also caused soil heavy metal hazards in agriculture. Heavy metals are extremely harmful to crops. These pollutants restrain seed germination, root elongation and whole-plant development, triggering stunting, leaf wilting and even plant mortality. Meanwhile, they damage photosynthetic structures, lower chlorophyll concentrations and net photosynthetic rates, thereby disturbing internal metabolism and degrading crop yield and commodity quality [87]. Targeted editing of the *BnCUP1* gene in rapeseed (*Brassica napus*) through CRISPR-Cas9 can significantly reduce cadmium accumulation in plants, while increasing biomass and yield in cadmium-contaminated farmland, without compromising normal growth and absorption of essential trace elements. The obtained *BnCUP1* edited line is a low-cadmium and high-yield rapeseed germplasm, addressing the problem of “cadmium reduction accompanied by yield loss” caused by editing cadmium transporter genes [77]. Two independent homozygous knockout lines of *WAKL4* were generated in *Arabidopsis thaliana* via CRISPR/Cas9 technology. Both lines exhibited pronounced cadmium-sensitive phenotypes, indicating that *WAKL4* functions as a key regulator of cadmium-specific tolerance in plants. This work provides a candidate genetic target for breeding low-cadmium Brassicaceae varieties [78]. Other CRISPR editing efforts targeted exon 7 of *BnaNRAMP1* to achieve “cadmium reduction without yield loss” in rapeseed and obtained the K140-22 low-cadmium and high-yield rapeseed germplasm resource [88]. Gene editing technology has successfully achieved precise regulation of cadmium accumulation in rapeseed (*Brassica napus*) and *Arabidopsis thaliana* by targeting and editing key genes such as *BnCUP1*, *WAKL4*, and *BnaNRAMP1*. Its core advantage lies in breaking through the bottleneck of “cadmium reduction accompanied by yield loss” in traditional cadmium reduction breeding, generating excellent germplasms with low cadmium, high yield, and unimpaired normal growth. However, current research remains largely limited to the single heavy metal type of cadmium stress, and the range of crop species to which it has been applied is narrow, and there are no reports on applications in other Brassicaceae crops or other heavy metals.

#### 3.2.5 Disease stress

Clubroot disease, caused by *Plasmodiophora brassicae*, is a soil-borne disease that specifically harms Brassicaceae crops such as Chinese cabbage, cabbage, and rapeseed, leading to root swelling, plant dwarfing, and severe yield reduction [79]. In China, this disease harms 3.2-4.0 million hectares of Brassicaceae crops every year, accounting for more than one-third of the total planting area of Brassicaceae crops in the country. It can cause an average yield reduction of 20%-30% in crops, and even total crop failure in severely affected fields. Its host range is wide, not only infecting major cultivated crops such as Chinese cabbage, cabbage, rapeseed, radish, and mustard, but also harming medicinal, ornamental and wild Brassicaceae plants such as *Isatis indigotica*, *Viola tricolor*, and *Eutrema wasabi* [89]. Using gene editing technology to improve the resistance of Brassicaceae crops to clubroot disease is crucial. Integrated assays combining genome-wide association analysis (GWAS) and CRISPR-Cas9 validated the major clubroot resistance locus *RPB1* in *Arabidopsis thaliana*, this work identified an unprecedented category of cation channel immune receptors and deepened our knowledge of plant basal immunity [90]. Other functional studies systematically analyzed the function of NLR-type clubroot resistance genes in *Brassica napus L*. using CRISPR-Cas9 technology, providing clear targets for creating broad-spectrum and durable disease-resistant germplasms through gene editing [91]. Additional CRISPR editing created new cabbage germplasms with simultaneous resistance to multiple diseases by knocking out susceptibility genes. *BoBPM6* is a susceptibility gene for black rot and clubroot in cabbage, and its knockout results in moderate resistance to both diseases, enriching the theoretical basis of the molecular mechanism of disease resistance in Brassicaceae crops [92]. The jasmonic acid (JA) signaling pathway regulates clubroot disease occurrence through the *BnaA05.MYC2-BnaA07.SUC2* module. Targeted knockout of *BnaA07.SUC2* via CRISPR-Cas9 greatly strengthens rapeseed resistance to clubroot, delivering a novel gene target for resistance breeding in Brassicaceae crops [93]. In addition to clubroot disease, CRISPR-Cas9 technology has also made notable progress in improving rapeseed resistance against *Sclerotinia sclerotiorum*. One study used a CRISPR/Cas9 multiplex system to edit two copies of *BnWRKY11* and four copies of *BnWRKY70* in *Brassica napus*. The resulting *BnWRKY70* mutants showed markedly stronger resistance to *S. sclerotiorum*, whereas plants overexpressing the gene were more susceptible, suggesting that *BnWRKY70* acts as a negative regulator of this resistance trait [94]. In a related study, the transcription factor *BnaA07.WRKY40* was found to be induced by *S. sclerotiorum.* Its protein product localizes to the nucleus, forms homodimers, and positively contributes to the immune response by binding directly to W-box motifs (TTGACT/C) in the promoters of downstream targets such as *HRP*, *BG2*, *PEP1*, and *ACNAT*. When this gene was knocked out using CRISPR/Cas9, mutant plants became significantly more vulnerable to the pathogen [95]. Another study knocked out *BnaPRT6*, a gene encoding an E3 ubiquitin ligase in the N-degron pathway. Loss of *BnaPRT6* function enhanced resistance to *S. sclerotiorum* through stabilization of *ERFVII* transcription factors. The level of resistance correlated positively with the number of copies mutated; the quadruple mutant, for instance, showed a 29.1% reduction in lesion area, with no apparent negative impact on yield or other key agronomic traits [80]. Taken together, these results indicate that gene editing can be useful not only for clubroot resistance but also for tackling fungal pathogens like *S. sclerotiorum*, offering both research opportunities and practical breeding potential. Concurrently, CRISPR/Cas9-mediated knockout of *GSL5* in *Brassica napus*, *Brassica rapa*, and *Brassica oleracea* was shown to confer broad-spectrum resistance against multiple pathotypes of *Plasmodiophora brassicae* by relieving jasmonic acid-mediated immune suppression, without compromising yield or agronomic traits, offering a valuable genetic resource for sustainable clubroot management in Brassicaceae crops [81].

Turnip mosaic virus (TuMV) is the most prevalent viral pathogen affecting Brassicaceae crops in China, significantly reducing both yield and quality of Brassicaceae crops. Aphids serve as the primary transmission vector for TuMV. As the most devastating viral disease affecting Brassicaceae crops, it causes 30%-70% yield losses globally, while causing a decline in vegetable quality. Typical symptoms include internal head necrosis in cabbage and failure to form heads in Chinese cabbage. Improving host resistance is the most effective and environmentally friendly means to prevent and control TuMV. Most mapped TuMV resistance genes remain uncharacterized at the functional level, with only genetic mapping completed [96]. CRISPR-Cas9-mediated site-directed editing was applied to target the host *eIF(iso)4E* gene, which encodes the eukaryotic translation initiation factor *eIF(iso)4E*. The resulting mutants exhibited complete immunity against Turnip mosaic virus (TuMV) with no observable developmental defects, providing a feasible technical approach for antiviral crop breeding [97]. CRISPR/Cas9-mediated editing of the *eIF4E* gene in *Arabidopsis thaliana* led to enhanced susceptibility to Turnip mosaic virus (TuMV), with a 3-fold increase in viral accumulation. The mutant plants exhibited severe developmental defects, furthering understanding of the *eIF4E* gene family [98]. The RNA polyadenylation factor *CPSF30* has been identified as a key host susceptibility factor for TuMV infection. CRISPR-mediated knockout of this gene in *Arabidopsis thaliana* drastically elevates plant resistance to TuMV, delivering valuable references for antiviral breeding across Brassicaceae crops [99]. *eIF2Bβ* has been identified as a key susceptibility gene for TuMV in Brassicaceae crops. Knockout of *eIF2Bβ* via CRISPR-Cas9 significantly enhances resistance to TuMV in mustard and *Brassica napus*, representing an efficient target for molecular breeding of antiviral Brassicaceae crops [100]. In *Brassica rapa* ssp*. pekinensis* (Chinese cabbage), *eIF(iso)4E* functions as an essential host factor for Turnip mosaic virus (TuMV) infection. CRISPR-Cas9-mediated targeted editing of *eIF(iso)4E* in Chinese cabbage generated edited plants with normal growth and development, no adverse phenotypes, and undetectable viral accumulation. This work develops a CRISPR-Cas9-based antiviral breeding approach for Chinese cabbage; the resulting resistant lines can serve as pre-breeding materials for disease-resistant variety development, and provide a reference for antiviral breeding of other Brassicaceae crops including radish, cabbage and rapeseed [101].

#### 3.2.6 Pest stress

Globally, pests such as aphids, rice stem borers, brown planthoppers, and fall armyworms are among the core factors causing yield reductions in Brassicaceae crops, and the yield losses they inflict account for a large share of overall losses from crop pathogens and herbivores. In Brassicaceae crops, primary pest control methods are chemical control, supplemented by crop rotation and physical control. However, due to the rapid evolution of pest resistance, the low level of scientific pesticide application among farmers, the lack of grassroots technical support and outdated application equipment, excessive pesticide use often results in poor pest control efficacy [102]. Therefore, improving the pest resistance of crops themselves is an emerging research direction. Glucosinolates (GS) are unique defensive secondary metabolites of Brassicaceae plants, and their hydrolysis products isothiocyanates (ITCs) have potent toxic activity on various pests and diseases. However, high glucosinolate levels in seeds will make the oil cake after oil extraction bitter and toxic, limiting its use as animal feed. It is critical to reduce seed glucosinolates while maintaining plant disease and pest resistance. Genome editing simultaneously targeted ten homologous copies of *GTR1* and *GTR2* in allotetraploid mustard (*Brassica juncea*), mutant seed GS concentrations fell below official double-low thresholds, meanwhile, GS levels in leaf margins and apices rose markedly, establishing a stronger defensive shield and improving tolerance to chewing herbivores [103]. *bHLH19* and *bHLH20* are novel negative regulators of the jasmonic acid (JA) pathway, simultaneous knockout of *bHLH19* and *bHLH20* in *Arabidopsis thaliana* via CRISPR Cas9 significantly enhanced plant resistance to fall armyworm and beet armyworm without compromising normal plant growth and development [104]. *BnaFAH* functions as a negative regulator of pest resistance in *Brassica napus* (rapeseed), and CRISPR-Cas9-mediated knockout of the *BnaFAH* gene enhanced resistance in *Brassica napus* to the major global agricultural pest *Plutella xylostella* under a 2-day short-day photoperiod. This resistance phenotype was achieved via activation of the reactive oxygen species (ROS)-jasmonic acid (JA) signaling cascade, providing a valuable candidate target and strategy for rapeseed pest resistance breeding [105].

CRISPR-Cas gene editing technology has become a core tool for enhancing plant disease and pest resistance. By knocking out susceptibility genes and precisely modifying resistance-associated loci, it enables the efficient development of new crop varieties with broad-spectrum resistance to viruses, fungi, oomycetes, bacteria and pests, providing key technical support for green and sustainable agriculture [106]. Practical genome editing research focused on insect-resistance breeding of Brassicaceae remains at an early stage, with only a limited number of peer-reviewed reports available. However, the scarcity of published pest resistance gene editing research in major cultivated Brassicaceae crops such as Chinese cabbage, cabbage, and radish, and the resulting research gap stands in stark contrast with the severe pest threats confronting the Brassicaceae crop industry, highlighting the urgent need for related research. Gene editing technology has shown great potential and broad application prospects in overcoming the bottlenecks of Brassicaceae pest resistance breeding.

## 4 Existing problems and prospects of gene editing research in Brassicaceae crops

CRISPR-Cas9 technology is widely used in Brassicaceae crops and plays an important role in genetic improvement and molecular breeding, but there are still several key limitations. This system has significant off-target effects. Cas9 can cleave DNA at non-target sites, leading to genome instability and disruption of normal gene functions. Although rational sgRNA design and the adoption of high-fidelity Cas9 variants can substantially reduce off-target events, such unintended genetic alterations cannot be fully eradicated under current technical conditions [107]. Current studies have shown that high-fidelity base editor *HFBE3* can be constructed via protein engineering, and a DNA-free ribonucleoprotein (RNP) protein delivery system can be established, which greatly improves the specificity of base editing and reduces off-target effects to a negligible level, laying a technical foundation for high-precision in vivo base editing [108]. Improving the efficiency of precise genome editing is also a crucial challenge. Homology-directed repair (HDR)-mediated genome editing can be used to precisely rewrite virtually any genomic locus and generate specified edits. Compared with non-homologous end joining (NHEJ), HDR supports precise genetic modifications including site-specific gene insertion and single-nucleotide substitution. Nevertheless, its extremely low efficiency in plant somatic cells remains the core constraint limiting large-scale deployment of precise plant genome editing [109]. Numerous studies have shown that the efficiency of HDR can be improved via strategies including geminivirus replicons, manipulating DNA repair pathways, and constructing plant gene targeting (GT) systems [[110], [111], [112]].

Cytosine base editors (CBE) and adenine base editors (ABE), as next-generation DNA base editing tools, enable single base modification at precise genomic loci. To date, CBEs have only been successfully applied in rapeseed (*Brassica napus*), while base editor-mediated precise genome editing remains largely unreported in other Brassicaceae crops, and most still rely on the non-homologous end joining (NHEJ) pathway to generate mutants [113]. However, conventional base editors are still restricted to C·G→T·A and A·T→G·C base transitions, and their editing efficiency varies significantly across different species and genomic loci. Currently, addressing off-target effects remains a critical and urgent challenge, and the efficiency of homology-directed repair (HDR)-mediated gene editing is still extremely low in plants. These limitations highlight an urgent demand for the development of high-sensitivity whole-genome off-target detection techniques and novel editing systems with improved specificity and editing accuracy.

Polyploidization is an important evolutionary feature of Brassicaceae plants, and the resulting genome complexity is a major obstacle limiting genetic improvement, in addition to off-target effects. Brassicaceae species have experienced a canonical whole-genome triplication (WGT) event. The inherent complexity of polyploid genomes therefore creates substantial challenges for high-quality genome sequencing and assembly [114]. Although polyploidization confers beneficial traits on crops such as functional redundancy, heterosis, and enhanced stress resistance, it also presents substantial challenges for genetic improvement due to the highly complex genome, the presence of a large number of homologous and homoeologous functional redundancy that makes it difficult to generate discernible phenotypes via single-gene mutations [115]. To date, the most widely adopted strategy is to design sgRNAs targeting conserved regions, enabling a single sgRNA to simultaneously target multiple homologous/homoeologous gene copies [116]. Owing to the complexity of the Brassicaceae genome, functional genomics research in Brassicaceae crops currently lags behind that in many other major crop species.

Establishing a stable and efficient genetic transformation system is a prerequisite for the large-scale deployment of gene editing in Brassicaceae crops. Currently, the most used delivery systems mainly include biolistic bombardment, *Agrobacterium*-mediated protoplast transformation, polyethylene glycol (PEG)-mediated method, viral vector method, and nanoparticle delivery system. Nevertheless, *Agrobacterium*-mediated transformation remains the most prevailing and practical approach for genetic modification in Brassicaceae species. Brassicaceae species are widely recognized as recalcitrant to regeneration, and their tissue culture recalcitrance is significantly higher than that of major field crops such as rice and maize. Transformant screening is challenging and associated with high false-positive rates. Traditional antibiotic screening (such as kanamycin, hygromycin) is highly toxic to Brassicaceae plants and severely impairs regeneration. Broccoli (*Brassica oleracea* var*. italica*) uses the *RUBY* betacyanin reporter gene to achieve visual screening of transformed calli with the naked eye, addressing the bottleneck of low transformation efficiency and difficult screening in broccoli, and eliminating the need for traditional antibiotic screening methods [117]. In non-heading Chinese (*Brassica rapa* var*. chinensis*) cabbage, using *Agrobacterium rhizogenes* K599 to co-express three developmental regulatory genes *ZmWUS2*, *IPT* and *AtPLT5* enabled direct regeneration of complete plants from callus without exogenous hormones, overcoming the long-standing challenge of extremely low transformation efficiency of non-heading Chinese cabbage [118]. However, traditional plant gene editing relies on tissue culture and *Agrobacterium* transformation, which has significant limitations. A major bottleneck of existing transformation systems is their significant genotype dependence, which makes it impossible to obtain efficient transformation for most crop varieties. Additionally, they also face technical problems such as cumbersome operation and long cycle, and somatic variation and epigenetic abnormalities are inevitably generated during the transformation process, further limiting their wide application in crop genetic improvement. Recent research has increasingly focused on tissue culture-free research to bypass the complex tissue culture stage. A relevant research team combined RNA mobile elements with viral delivery systems, overcoming the limitation that virus-mediated gene editing cannot efficiently enter germ cells, enabling tissue culture-free editing [119]. A tissue culture-free virus-mediated gene editing system in sorghum using foxtail mosaic virus (FoMV) enhanced somatic cell editing efficiency [120]. Nanoparticles, owing to their unique physicochemical properties, are expected to solve the bottleneck of biomolecule delivery in plants and establish a universal, efficient, and non-invasive genetic engineering platform, but their application in plants remains in its infancy [121]. Overall, the genetic transformation bottleneck of the vast majority of Brassicaceae crops has not been fundamentally solved, and the overall level lags far behind field crops such as rice and corn. Significant efforts are still required to achieve efficient genetic transformation of Brassicaceae crops.

Looking ahead, multiplex and multi-trait stacking editing is expected to become an important direction in precision breeding of Brassicaceae crops. Although CRISPR-Cas9 has already enabled targeted improvement of single genes or homologous copies, simultaneous optimization of multiple complex traits—such as plant architecture, disease resistance, drought tolerance, quality, and yield—still requires coordinated manipulation of several unlinked loci. Recent progress in multiplex CRISPR systems, including tRNA-sgRNA and Cpf1-mediated editing, has laid the technical groundwork for co-editing multiple targets in a single transformation event. Yet, further advances are needed in three areas: editing tools, design strategies, and transformation systems.

At the tool level, high-sensitivity whole-genome off-target detection methods are becoming more robust, offering improved support for safety assessments in multi-gene editing. AI-assisted sgRNA design platforms now allow in silico evaluation of different gene combinations before experiments begin, reducing unnecessary trial and error. The use of tissue-specific or inducible promoters can also help confine editing events to particular tissues or developmental stages, thereby minimizing unintended interference with normal plant growth. In addition, newer tools such as base editors and prime editors are expanding the scope of genome modification, with the potential to achieve more precise genetic changes than conventional knockout approaches.

At the strategic level, the emphasis should not be on the sheer number of genes edited, but on selecting the right targets. By integrating multi-omics data and constructing regulatory networks for target traits, it becomes possible to identify hub genes that influence multiple downstream effects. Prioritizing such nodal genes for editing may allow simultaneous improvement of several traits with fewer modifications. Meanwhile, the increasing application of machine learning could help predict phenotypic outcomes based on editing combinations, making experimental design more targeted.

At the transformation level, tissue-culture-free systems offer a promising way to bypass genotype restrictions and reduce turnaround time. Nanomaterial-based delivery methods are relatively simple to operate and cause less damage to plant tissues. Transient co-expression of regeneration-related genes has already shown beneficial effects in some recalcitrant crop species. Further integration and refinement of these three approaches are expected to gradually lower the barriers and costs associated with creating multi-gene edited materials.

Overall, as tools, strategies, and transformation systems continue to mature, breeding approaches for Brassicaceae crops may shift from trait-by-trait improvement toward multi-trait synergistic design. In the future, a single editing event could potentially improve plant architecture, stress resistance, quality, and yield simultaneously, providing a new route to develop varieties with comprehensively improved agronomic performance.

## 5 Conclusion

Gene editing technology has greatly promoted the genetic improvement of Brassicaceae crops by virtue of its high precision, efficiency and target specificity. Gene editing technology should be combined with high-throughput phenotyping analysis, genomics, speed breeding, artificial intelligence (AI) and other technologies to further accelerate the application of gene editing in Brassicaceae crops. Gene editing technology has achieved breakthrough progress in conferring broad-spectrum resistance to clubroot disease, reducing heavy metal accumulation, and developing male sterile lines in Brassicaceae. These advances provide an indispensable technical approach for solving core bottlenecks in traditional breeding, such as the narrow resistance gene pool and the difficulty of harnessing functional redundancy in polyploid genomes. However, it still faces systemic challenges such as high genotype dependence of genetic transformation, low efficiency of multiplex editing of homologous/homoeologous gene copies, scarcity of research in industrially relevant areas such as pest resistance, and inadequate field performance stability evaluation and long-term biosafety assessment frameworks. Consequently, significant gaps remain before the large-scale application of gene editing breeding across all Brassicaceae species and for multiple agronomic traits. Despite regulatory and ethical challenges, gene editing continues to offer tremendous potential for the genetic improvement of Brassicaceae crops.

## Author contributions

Xiaolong Wang and Bingbing Shangguan collected and sorted literatures, drafted the manuscript, and prepared figures and tables. Yanwen Zhang, Tongbing Su, Jialei Ji and Jianjun Zhao assisted with manuscript revisions. Tongkun Liu, Xilin Hou, Ying Li, and Changwei Zhang supervised the whole work and revised and finalized the full manuscript.

## Declaration of competing interest

The authors declare that they have no known competing financial interests or personal relationships that could have appeared to influence the work reported in this paper.
